# PROMs als Instrument der Ergebnisbewertung nach primärer Knieendoprothese in drei unterschiedlichen EndoCert-zertifizierten Versorgungsstrukturen

**DOI:** 10.1007/s00132-026-04824-x

**Published:** 2026-04-14

**Authors:** Katrin Osmanski-Zenk, Jan Masanneck, Philipp Bergschmidt, Claus Köttgen, Wolfram Mittelmeier, Martina Rohde-Lindner, Annett Klinder

**Affiliations:** 1 Orthopädische Klinik und Poliklinik, Universitätsmedizin Rostock, Doberaner Straße 142, 18057 Rostock, Deutschland; 2 Klinik für Orthopädie und Unfallchirurgie, Klinikum Südstadt Rostock, Rostock, Deutschland; 3 Orthopädische Praxis Dr. Köttgen, Hagenow, Deutschland

**Keywords:** Patient-reported outcome measures (PROMs), Knieendoprothetik, Versorgungsstruktur, Qualitätsbewertung, Ergebnisqualität, Patient-reported outcome measures (PROMs), Total knee arthroplasty, Institutional surgical setting, Quality assessment, Outcome quality

## Abstract

**Hintergrund:**

Patientenberichtete Ergebnismessungen (PROM) werden in Deutschland bislang nur eingeschränkt genutzt. Für EndoCert-Zentren besteht keine verpflichtende PROM-Erhebung. Zudem fehlen Analysen, die PROM-Ergebnisse systematisch mit klinischen Nachuntersuchungen und der Beurteilung des Behandlungserfolges verknüpfen. Ziel war es, den Zusammenhang zwischen PROM und der klinisch definierten Behandlungszielerreichung 3 Monate nach Knie-TEP zu untersuchen und zu prüfen, ob sich Unterschiede zwischen verschiedenen EndoCert-zertifizierten Versorgungsstrukturen zeigen.

**Material und Methoden:**

In einer prospektiven multizentrischen Studie zum Potenzial von PROM in der Endoprothetik wurden Patienten nach Knie-TEP in einer Universitätsmedizin, einem Klinikum und einer Praxis mit Anbindung an ein Endoprothetikzentrum eingeschlossen. Drei Monate postoperativ erfolgte die Erhebung von PROM (Oxford Knee Score, EuroQol-5D, EndoCert-Risk-Scores) und eine klinische Nachuntersuchung mit Bewertung des Behandlungsergebnisses. Die PROM-Ergebnisse wurden zwischen Patienten mit abgeschlossener Behandlung und solchen mit weiterem Nachsorge- oder Behandlungsbedarf sowie zwischen den Zentrumstypen verglichen.

**Ergebnisse:**

Patienten mit erreichtem Behandlungsziel wiesen signifikant bessere postoperative PROM-Werte auf als jene mit weiterem Nachsorgebedarf (*p* < 0,001). Zwischen den Zentrumsstrukturen zeigten sich keine schmerzbezogenen Unterschiede. In der Gruppe „weitere Behandlung erforderlich“ bestanden signifikante zentrumsabhängige Unterschiede in den funktions- und lebensqualitätsbezogenen PROM.

**Diskussion:**

PROM spiegeln das Outcome nach Knie-TEP zuverlässig wider und unterstreichen ihr Potenzial als Ergänzung der Ergebnisbewertung im Rahmen von EndoCert und EPRD. Die Ergebnisse sprechen für eine strukturierte Nutzung als Frühwarnsystem zur gezielten Nachsorgesteuerung unter Berücksichtigung patienten- und zentrumsspezifischer Faktoren.

**Graphic abstract:**

**Figure:**
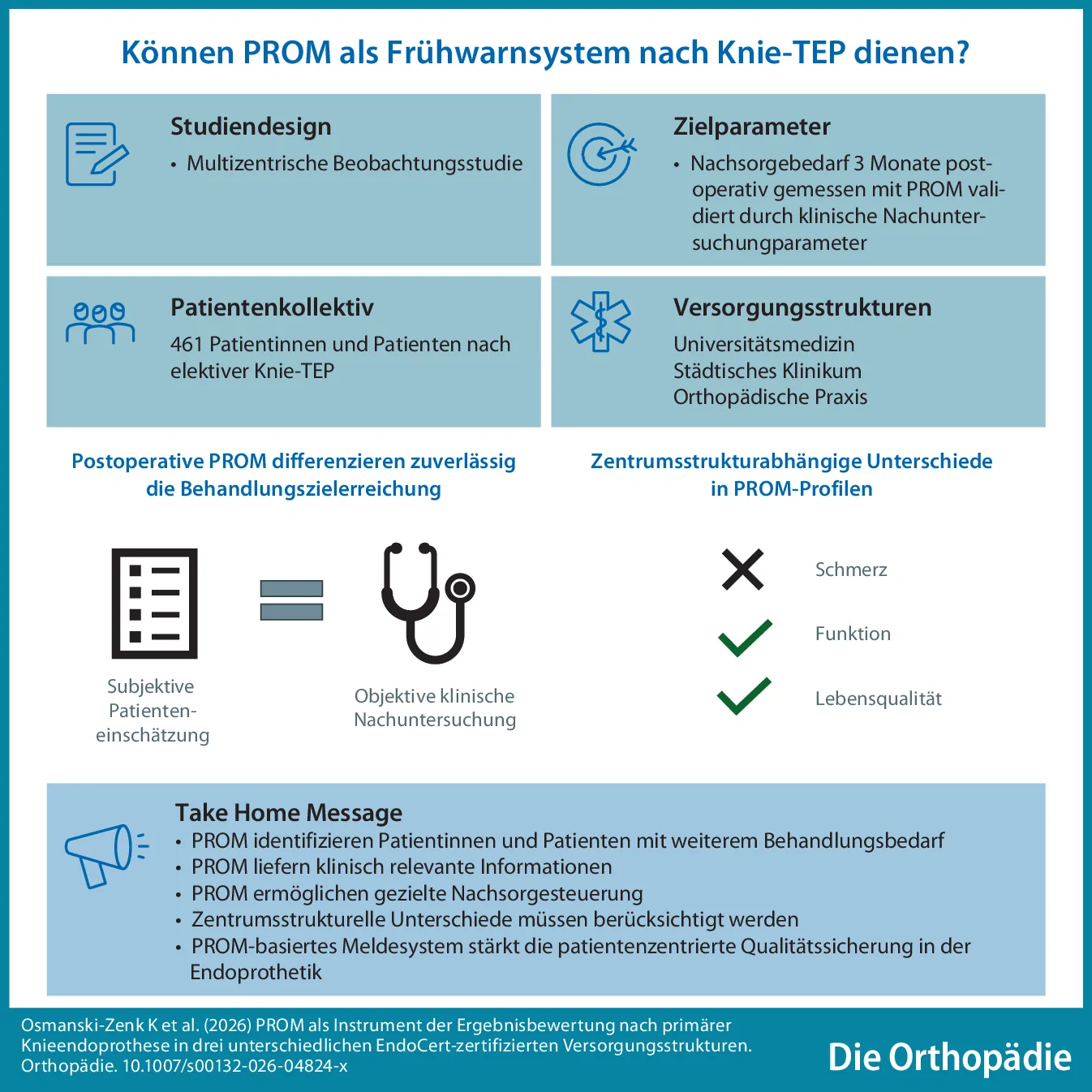

## Abstract (en)

**Background:**

The use of patient-reported outcome measures (PROMs) is still limited in Germany. For EndoCert-certified centers, PROM collection is not mandatory, and analyses systematically linking PROM results with clinical follow-up examinations and the assessment of treatment success are lacking. The aim of this study was to investigate the association between PROMs and clinically defined achievement of treatment goals 3 months after total knee arthroplasty (TKA) and to assess whether differences exist between various EndoCert-certified institutional surgical settings.

**Materials and methods:**

In a prospective multicenter study evaluating the potential of PROMs in arthroplasty, patients undergoing TKA were included at a university hospital, a general hospital, and an outpatient practice affiliated with an arthroplasty center. Three months postoperatively, PROMs (Oxford Knee Score, EuroQol-5D, EndoCert risk scores) were collected and a clinical follow-up examination was performed to assess achievement of treatment goals. PROM results were compared between patients with achieved treatment goals and those who required further treatment, as well as among the different center types.

**Results:**

Patients who achieved the defined treatment goals showed significantly better postoperative PROM scores than those who did not (*p* < 0.001). No pain-related differences were observed between the different institutional surgical settings. In contrast, among patients with unmet treatment goals, significant center-dependent differences were found in function- and quality-of-life-related PROMs.

**Discussion:**

PROMs reliably reflect outcomes after TKA and highlight their potential as a complementary tool for outcome assessment within the frameworks of EndoCert and the German Arthroplasty Registry. The findings support a structured use of PROMs as an early warning system for targeted postoperative care management, taking patient- and center-specific factors into account.

## Hintergrund

Patientenberichtete Ergebnismessungen („patient-reported outcome measures“ [PROM]) haben sich international als zentrale Instrumente der Ergebnisbewertung nach endoprothetischen Eingriffen etabliert. Nationale Endoprothesenregister in Ländern wie Schweden, dem Vereinigten Königreich und Australien erfassen PROM seit vielen Jahren routinemäßig prä- und postoperativ und nutzen diese Daten nicht nur zur wissenschaftlichen Auswertung, sondern auch zur kontinuierlichen Qualitätsmessung, zum Benchmarking von Kliniken sowie zur Unterstützung der gemeinsamen Entscheidungsfindung mit Patienten. Darüber hinaus leisten PROM-basierte Registerdaten einen Beitrag zum systematischen Evidenznachweis und ermöglichen die Identifikation erfolgreicher Versorgungsansätze und Behandlungsstrategien [1, 25, 28].

Die systematische Auswertung von PROM-Daten in internationalen Registern hat Erkenntnisse zur Anwendung bei der klinischen Versorgung geliefert. So konnten relevante Unterschiede in der Ergebnisqualität zwischen Patientengruppen, Behandlungsstrategien, Operationsverfahren und Implantattypen identifiziert werden, was zur Weiterentwicklung von Indikationsstellungen, Nachsorgekonzepten und qualitätsorientierten Versorgungsmodellen beigetragen hat [19, 25]. Darüber hinaus zeigen Langzeitdaten, dass individuelle Patientenfaktoren wie Alter, Komorbiditäten, präoperatives Funktionsniveau und psychische Gesundheit den subjektiven Nutzen einer Endoprothesenimplantation maßgeblich beeinflussen [2, 13, 23, 24]. In einigen Gesundheitssystemen sind PROM daher fester Bestandteil klinischer Behandlungspfade und dienen unter anderem als Frühwarninstrument bei reduziertem postoperativem Behandlungsergebnis [25].

In deutschen Versorgungsstrukturen werden PROM in der Endoprothetik bislang nur eingeschränkt genutzt. Mit Einführung des EndoCert-Zertifizierungssystems im Jahr 2012 wurde die Erhebung von PROM empfohlen, eine verpflichtende Vorgabe besteht jedoch bis heute nicht [8, 9]. Die Qualitätssicherung fokussiert sich bislang überwiegend auf Struktur- und Prozessmerkmale, radiologische Indikatoren, Komplikationen sowie revisionsbasierte Endpunkte. Seit 2025 eröffnet die Möglichkeit der PROM-Erfassung über das Endoprothesenregister Deutschland (EPRD) erstmals eine zentral organisierte und ressourcenschonende Erhebung, bei der wesentliche Aufgaben durch das Register übernommen werden. Dennoch bleibt die Teilnahme an der PROM-Erhebung freiwillig, sodass eine flächendeckende Implementierung patientenzentrierter Ergebnisparameter bislang nicht gewährleistet ist [2–4]. Allerdings wurden mit dem Beschluss des Gemeinsamen Bundesausschusses (G-BA) zum Projekt „PROMoting Quality“ in Deutschland konkrete Schritte zur systematischen Einführung patientenberichteter Outcomes in die Qualitätsbewertung der Endoprothetik eingeleitet [11].

Im Zusammenhang mit dem flächendeckenden Einsatz von PROM in der Endoprothetik stellt die Validierung von PROM-Ergebnissen durch klinische Parameter einen bislang unzureichend adressierten Aspekt dar. PROM erfassen subjektive Wahrnehmungen von Patientenzufriedenheit, Schmerz, Funktion und Lebensqualität, deren klinische Relevanz jedoch erst durch die systematische Gegenüberstellung mit objektiven klinischen Befunden und der Behandlungszielerreichung vollständig eingeordnet werden kann. Erste Studien konnten zeigen, dass PROM zur Identifikation potenzieller Risikopatienten nach Versorgung mit einer primären Knietotalendoprothese (Knie-TEP) in einer EndoCert-zertifizierten Einrichtung geeignet sind [18]. Es fehlen jedoch multizentrische Analysen, die PROM mit standardisierten klinischen Nachuntersuchungen verknüpfen und dabei unterschiedliche Versorgungsstrukturen berücksichtigen. Das EndoCert-Zertifizierungssystem unterscheidet grundsätzlich zwischen Endoprothetikzentren (EPZ) und Endoprothetikzentren der Maximalversorgung (EPZmax). Darüber hinaus können u. a. operative Einheiten wie orthopädische Praxen oder Belegarztstrukturen an ein EPZ oder EPZmax angebunden sein und innerhalb dieser Versorgungsstruktur endoprothetische Leistungen erbringen [9]. In der vorliegenden Studie wurden eine Universitätsmedizin als EPZmax, ein städtisches Klinikum als EPZmax sowie eine an ein EPZ angebundene orthopädische Praxis betrachtet.

### Ziel der Arbeit

Vor diesem Hintergrund untersucht die vorliegende Arbeit PROM nach primärer Knie-TEP in drei nach EndoCert zertifizierten Einrichtungen dieser drei unterschiedlichen Versorgungsstrukturen. Neben der Erhebung postoperativer PROM erfolgte 3 Monate postoperativ eine standardisierte klinische Nachuntersuchung mit Bewertung der Behandlungszielerreichung. Ziel der Analyse war es, den Zusammenhang zwischen PROM-Ergebnissen und klinischem Outcome darzustellen und zu prüfen, ob sich hierbei Unterschiede zwischen den verschiedenen EndoCert-zertifizierten Zentrumsstrukturen zeigen.

Die Ergebnisse dieser Studie sollen dazu beitragen, die Rolle von PROM als patientenzentrierte Ergänzung zur Ergebnisbewertung in der Endoprothetik in Deutschland einzuordnen und deren Potenzial für eine differenzierte Nachsorgesteuerung unter Berücksichtigung ressourcenschonender Versorgungsstrukturen kritisch zu beleuchten.

## Material und Methoden

### Studiendesign und Kollektiv

Diese Arbeit basiert auf einer prospektiven, multizentrischen Beobachtungsstudie in drei nach EndoCert zertifizierten Einrichtungen unterschiedlicher Versorgungsstruktur. Die Studie knüpft in Design und Methodik an Studien zur Identifikation potenzieller Risikopatienten anhand von PROM an [17, 18]. Die Studie wurde durch die zuständige Ethikkommission genehmigt.

Alle Studienteilnehmenden wurden vollumfänglich über Zielsetzung, Ablauf und Datenschutz informiert und gaben ihre schriftliche Einwilligung. An der Orthopädischen Universitätsmedizin und der Orthopädischen Klinik erfolgte die Aufklärung im Rahmen der Indikationsstellung, während in der orthopädischen Praxis die Studienteilnehmenden im Zuge der postoperativen Nachsorge 3 Monate nach dem Eingriff rekrutiert wurden. In den klinischen Einrichtungen wurden die PROM (EQ-5D-3L, EndoCert-Riskscore, Oxford Knee Score) 10–11 Wochen postoperativ und in der Praxis 3 Monate postoperativ erhoben. Zusätzlich erfolgte 3 Monate postoperativ eine standardisierte klinische Nachuntersuchung zwischen der 12. und 16. Woche nach Operation. Basierend auf vorangegangenen Studienergebnissen [17], ist die Eignung postoperativer PROM zur Identifikation potenzieller Risikopatienten bereits belegt. Die standardisierte klinische Nachuntersuchung erfolgte in der Praxis durch den Operateur selbst, während sie in den klinischen Einrichtungen durch geschulte wissenschaftliche Mitarbeitende unter fachärztlicher Supervision oder unterschiedlichen orthopädischen Fachärzten, die nicht zwingend am operativen Eingriff beteiligt waren, durchgeführt wurde.

**Abb. 1:**
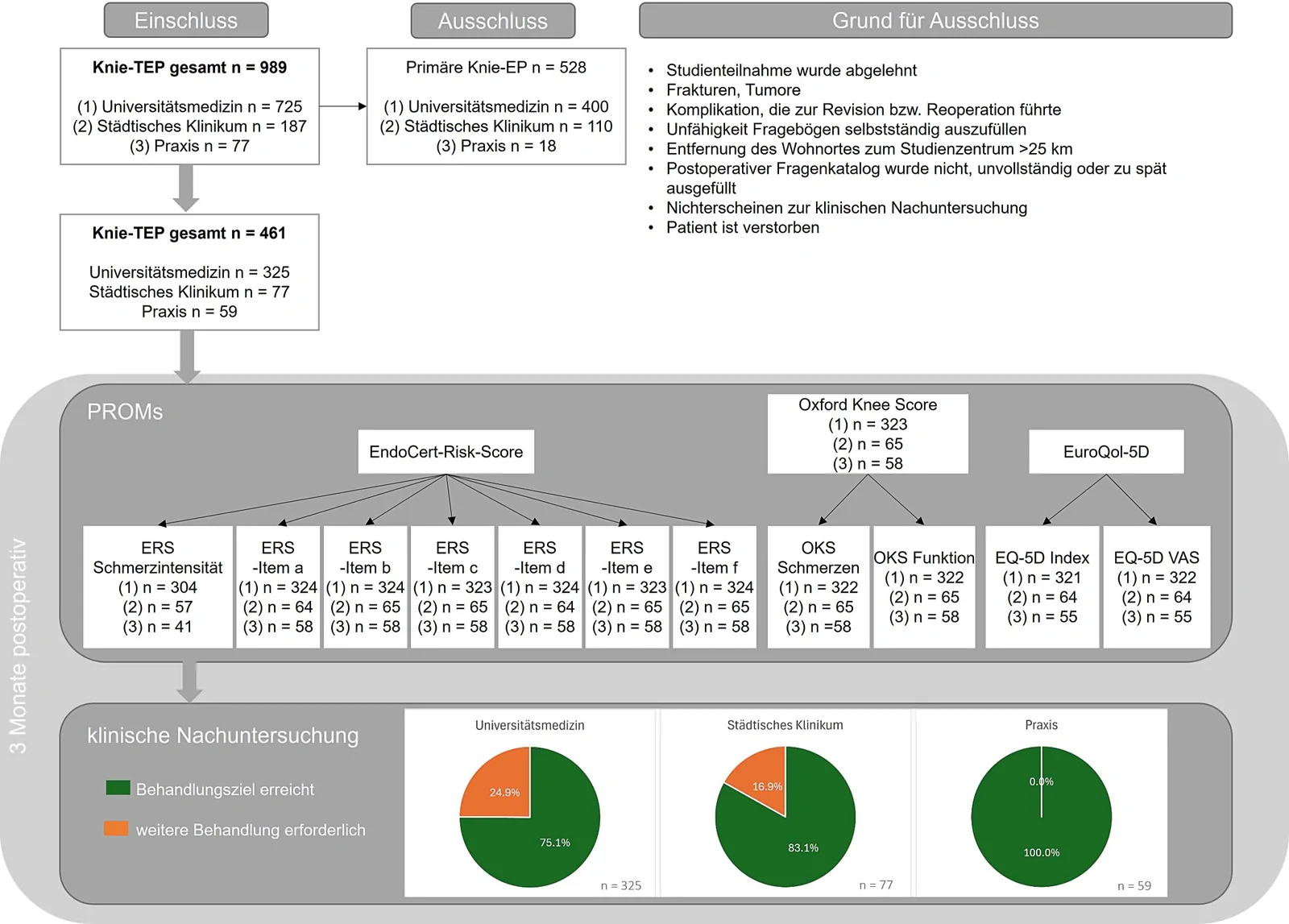
Einschluss, Datenerhebung, Nachbeobachtung und Datenanalyse. *TEP *Totalendoprothese, *n *Anzahl der Fälle, *PROM *„patient-reported outcome measures“, *Item a: *Hatten Sie während der letzten 4 Wochen beim Gehen Schmerzen in Ihrem betroffenen Gelenk? *Item* *b: *Hatten Sie während der letzten 4 Wochen nachts Knieschmerzen, sodass Sie nicht schlafen konnten? *Item c: *Verglichen mit meinem allgemeinen Gesundheitszustand während der vergangenen 12 Monate ist mein heutiger Gesundheitszustand besser? *Item d: *Würden Sie die Gelenkoperation aus heutiger Sicht erneut durchführen lassen? *Item e: *Würden Sie die bei Ihnen durchgeführte Gelenkoperation einer anderen Person empfehlen? *Item f: *Würden Sie das EndoProthetikZentrum, in dem Ihre Gelenkoperation durchgeführt wurde, weiterempfehlen? *VAS *visuelle Analog-Skala, *ERS *EndoCert-Riskscore, *OKS *Oxford Knee Score, *EQ-5D *Euroqol-5-Dimensionen

Insgesamt wurden 461 Patienten aus den drei EndoCert-zertifizierten Einrichtungen in die Analyse eingeschlossen (Abb. 1). In der Orthopädischen Klinik der Universitätsmedizin wurden zwischen März 2017 und Januar 2023 nach Anwendung der Ein- und Ausschlusskriterien 325 Fälle berücksichtigt, im städtischen Klinikum zwischen März 2022 und April 2023 77 Fälle sowie in der angebundenen orthopädischen Praxis zwischen Januar 2022 und Mai 2023 59 Fälle. Bei drei Patienten wurden im Beobachtungszeitraum bilateral primäre Knieendoprothesen implantiert, sodass beide Eingriffe jeweils als eigenständige Fälle in die Auswertung eingingen.

### Erhobene PROM und klinische Nachuntersuchung

Zur patientenzentrierten Ergebnisbewertung wurden validierte, krankheitsspezifische als auch generische PROM eingesetzt. Der Oxford Knee Score (OKS) wurde zur Erfassung gelenkspezifischer Beschwerden nach Knie-TEP verwendet und erlaubt eine differenzierte Bewertung der Subdimensionen Schmerz und Funktion [10, 16]. Ergänzend wurde mit dem EuroQol-5D-3L (EQ-5D-3L) ein generisches Instrument zur Erfassung der gesundheitsbezogenen Lebensqualität eingesetzt [7]. Der EndoCert-Riskscore (ERS), bislang nicht validiert, erfasst patientenberichtete Schmerzintensität, funktionelle Einschränkungen, den subjektiven Gesundheitszustand sowie Aspekte der Patientenzufriedenheit, einschließlich der Weiterempfehlungsbereitschaft von Operation und Endoprothetikzentrum [18].

Im Rahmen der standardisierten klinischen Nachuntersuchung wurde durch den Untersuchenden anhand definierter anamnestischer und klinischer Parameter beurteilt, ob eine Konsequenz aus der Nachuntersuchung resultiert. Zu den erhobenen Nachuntersuchungsparametern zählten die Beurteilung des Gangbildes, das Erfassen einer Beinlängendifferenz, das Bewegungsausmaß nach der Neutral-Null-Methode, die Gehfähigkeit ohne Gehstützen, die maximale Gehstrecke sowie Schmerzen beim Gehen und in Ruhe. Darüber hinaus wurden Wundheilungsstörungen und Narbenverhältnisse dokumentiert, ebenso die Einnahme von Schmerzmitteln, die Schmerzursache, das Vorliegen einer Gelenkinstabilität sowie das Zohlen-Zeichen. Auf Grundlage dieser Parameter erfolgte die Zuordnung, ob aus klinischer Sicht keine weitere Nachsorge erforderlich war oder ob zusätzlicher diagnostischer, therapeutischer oder operativer Behandlungsbedarf bestand. Patienten, bei denen in der Nachuntersuchung keine Indikation für weitere diagnostische und therapeutische Maßnahmen vorlag, wurden der Gruppe „Behandlungsziel erreicht“ zugeordnet, während eine weiterführende Nachsorge, zusätzliche diagnostische Maßnahmen, eine physiotherapeutische Behandlung oder eine Revisionsindikation eine Zuordnung zur Gruppe „weitere Behandlung erforderlich“ bedingten.

### Statistische Analyse

Die statistische Auswertung erfolgte mit SPSS® (IBM®, Ehningen, Deutschland) in der Version 29.0.1.1. Das Signifikanzniveau wurde auf *p* < 0,05 festgelegt. Analysiert wurden zum einen Unterschiede zwischen den Gruppen „Behandlungsziel erreicht“ und „weitere Behandlung erforderlich“, zum anderen zentrumsbezogene Unterschiede innerhalb dieser Gruppen, um die Auswirkung der jeweiligen Versorgungsstruktur bewerten zu können. Da die Prüfung der metrischen Variablen mittels Shapiro-Wilk-Test keine Normalverteilung ergab, wurden nichtparametrische Verfahren angewendet [22]. Für Vergleiche zwischen zwei Gruppen kam der Mann-Whitney-U-Test (U-Test), bei Vergleichen zwischen mehr als zwei Zentren der Kruskal-Wallis-Test (H-Test) zum Einsatz [14, 15]. Ordinal, nominal und binär skalierte Variablen wurden mithilfe des Chi-Quadrat-Tests auf Unabhängigkeit analysiert, bei Verletzung der Testvoraussetzungen wurde der exakte Test nach Fisher verwendet [6, 20].

## Ergebnisse

Die Basischarakteristika der Studienpopulation sind in Tab. 1 dargestellt. Zwischen den Studienteilnehmenden der Universitätsmedizin und der Praxis zeigte sich ein signifikanter Unterschied (H-Test, *p* = 0,031) im Charlson-Komorbiditäts-Index, wobei die höheren Werte an der Orthopädischen Universitätsmedizin auf eine höhere Komorbiditätslast im Patientenkollektiv hinweisen.

**Tab. 1:** Basischarakteristika der Studienpopulation bei Knie-TEP in den drei Zentren (Universitätsmedizin, städtisches Klinikum und Praxis)

		Universitätsmedizin	Städtisches Klinikum	Orthopädische Praxis	*p*-Wert^d^
Gesamt		325	77	59	/
Geschlecht	Männlich (*n* (%))	159 (48,9)	32 (41,6)	30 (50,8)	0,454^d^
	Weiblich (*n* (%))	166 (51,1)	45 (58,4)	29 (49,2)	
Alter bei Operation (Jahre) (Mittelwert [SD])		66,93 (9,54)	70,4 (8,95)	67,49 (10,77)	0,271^d^
BMI (kg/m^2^) (Mittelwert [SD])		31,26 (5,48)	31,28 (5,87)	32, 61 (5,85)	0,227^d^
Erneuerter Charlson-Index (Median [R])		0 (‑7-23)^1^	0 (‑7-23)	0 (‑7-5)^1^	*0,030*^*d*^
Van Walraven-Elixhauser (Median [R])		0 (0–4)	0 (0–6)	0 (0–2)	0,219^d^

^d^Kruskal-Wallis-Test, ^1^ signifikanter Gruppenunterschied

*BMI *Body-Mass-Index,* n* Anzahl, *SD* Standardabweichung, *R* Range

Bei der gruppenbezogenen Analyse nach Behandlungszielerreichung ist zu berücksichtigen, dass in der Praxis keine Patienten der Gruppe „weitere Behandlung erforderlich“ zugeordnet wurden, sodass hierfür keine vergleichenden Auswertungen möglich waren (Abb. 1). In den beiden übrigen Zentren zeigte sich ein signifikanter Altersunterschied zwischen den Gruppen, wobei Patienten der Gruppe „weitere Behandlung erforderlich“ signifikant jünger (4 Jahre in der Universitätsmedizin, U‑Test, *p* = 0,036; 7 Jahre im städtischen Klinikum, U‑Test, *p* = 0,010) waren als jene mit erreichter Zielerfüllung. Zudem bestand in der Orthopädischen Universitätsmedizin ein signifikanter Unterschied im Body-Mass-Index (U-Test, *p* = 0,008), wobei der BMI in der Gruppe „weitere Behandlung erforderlich“ im Mittel um etwa 5 Punkte höher lag als in der Gruppe „Behandlungsziel erreicht“.

Grundsätzlich zeigten sich zwischen den Gruppen „Behandlungsziel erreicht“ und „weitere Behandlung erforderlich“ in der Universitätsmedizin, im städtischen Klinikum sowie in der Gesamtpopulation für alle erhobenen PROM hochsignifikante Unterschiede (jeweils *p* < 0,001).

### Assoziation der postoperativen Patientenzufriedenheit mit der Behandlungszielerreichung in Abhängigkeit von der Versorgungsstruktur

Die ERS-Items zur erneuten Operationsbereitschaft (d), Weiterempfehlung der Operation (e) und Weiterempfehlung des Zentrums (f) waren jeweils hochsignifikant mit der Behandlungszielerreichung assoziiert (alle *p* < 0,001). Eine zentrumsunabhängige und somit konsistente Assoziation zwischen postoperativer Patientenzufriedenheit und Outcome zeigte sich jedoch nur im ERS-Item f hinsichtlich der Weiterempfehlung des Zentrums (Tab. 2).

**Tab. 2:** Die Items d, e und f des ERS zur postoperativen Evaluierung der Patientenzufriedenheit zwischen den beiden Gruppen „Behandlungsziel erreicht“ und „weitere Behandlung erforderlich“ unterschieden nach den drei Versorgungsstrukturen

Score	Zentrum	Behandlungsziel erreicht *n* (%)					Weitere Behandlung erforderlich *n* (%)					*p*-Wert^B^
		1	2	3	4	5	1	2	3	4	5	
ERS-Item d^1^	Universitätsmedizin	153 (63,0)	63 (25,9)	15 (6,2)	9 (3,7)	3 (1,2)	30 (37,0)	19 (23,5)	17 (21,0)	12 (14,8)	3 (3,7)	*<* *0,001*^*a*^
	Städtisches Klinikum	33 (62,3)	14 (26,4)	6 (11,3)	0 (0,0)	0 (0,0)	0 (0,0)	3 (27,3)	2 (18,2)	3 (27,3)	3 (27,3)	*<* *0,001*^*a*^
	Orthopädische Praxis	35 (60,3)	15 (25,9)	5 (8,6)	2 (3,4)	1 (1,7)	–	–	–	–	–	–
	*p‑Wert*^*S*^	0,806^a^					*0,010*^*a*^					–
	Gesamt	221 (62,4)	92 (26,0)	26 (7,3)	11 (3,1)	4 (1,1)	30 (32,6)	22 (23,9)	19 (20,7)	15 (16,3)	6 (6,5)	*<* *0,001*^*a*^
ERS-Item e^2^	Universitätsmedizin	142 (58,7)	71 (29,3)	21 (8,7)	5 (2,1)	3 (1,2)	26 (32,1)	29 (35,8)	17 (21,0)	8 (9,9)	1 (1,2)	*<* *0,001*^*a*^
	Städtisches Klinikum	35 (64,8)	15 (27,8)	3 (5,6)	1 (1,9)	0 (0,0)	1 (9,1)	2 (18,2)	3 (27,3)	2 (18,2)	3 (27,3)	*<* *0,001*^*a*^
	Orthopädische Praxis	40 (69,0)	10 (17,2)	2 (3,4)	2 (3,4)	4 (6,9)	–	–	–	–	–	–
	*p‑Wert*^*S*^	0,064^a^					*<* *0,001*^*a*^					–
	Gesamt	217 (61,3)	96 (27,1)	26 (7,3)	8 (2,3)	7 (2,0)	27 (29,3)	31 (33,7)	20 (21,7)	10 (10,9)	4 (4,3)	*<* *0,001*^*a*^
ERS-Item f^3^	Universitätsmedizin	186 (76,5)	49 (20,2)	7 (2,9)	1 (0,4)	0 (0,0)	41 (50,6)	22 (27,2)	13 (16,0)	4 (4,9)	1 (1,2)	*<* *0,001*^*a*^
	Städtisches Klinikum	42 (77,8)	11 (20,4)	1 (1,9)	0 (0,0)	0 (0,0)	2 (18,2)	5 (45,5)	2 (18,2)	2 (18,2)	0 (0,0)	*<* *0,001*^*a*^
	Orthopädische Praxis	47 (81,0)	9 (15,5)	2 (3,4)	0 (0,0)	0 (0,0)	–	–	–	–	–	–
	*p‑Wert*^*S*^	0,966^a^					0,195^a^					–
	Gesamt	275 (77,5)	69 (19,4)	10 (2,8)	1 (0,3)	0 (0,0)	43 (46,7)	27 (29,3)	15 (16,3)	6 (6,5)	1 (1,1)	*<* *0,001*^*a*^

^a^Chi-Quadrat-Test, *p*-Wert^S^ = Vergleich Studienzentren, *p*-Wert^B^ = Vergleich Behandlungsziel

^1^Würden Sie die Gelenkoperation aus heutiger Sicht erneut durchführen lassen? (1 = ja, sicher, 2 = wahrscheinlich ja, 3 = unsicher, 4 = wahrscheinlich nicht, 5 = sicher nicht); ^2^Würden Sie die bei Ihnen durchgeführte Gelenkoperation einer anderen Person empfehlen? (1 = ja, sicher, 2 = wahrscheinlich ja, 3 = unsicher, 4 = wahrscheinlich nicht, 5 = sicher nicht); ^3^Würden Sie das EndoProthetikZentrum, in dem Ihre Gelenkoperation durchgeführt wurde, weiterempfehlen? (1 = ja, sicher, 2 = wahrscheinlich ja, 3 = unsicher, 4 = wahrscheinlich nicht, 5 = sicher nicht); *ERS* EndoCert-Risk-Score, *n* Anzahl

### Assoziation der postoperativen Schmerzen mit der Behandlungszielerreichung in Abhängigkeit von der Versorgungsstruktur

**Abb. 2:**
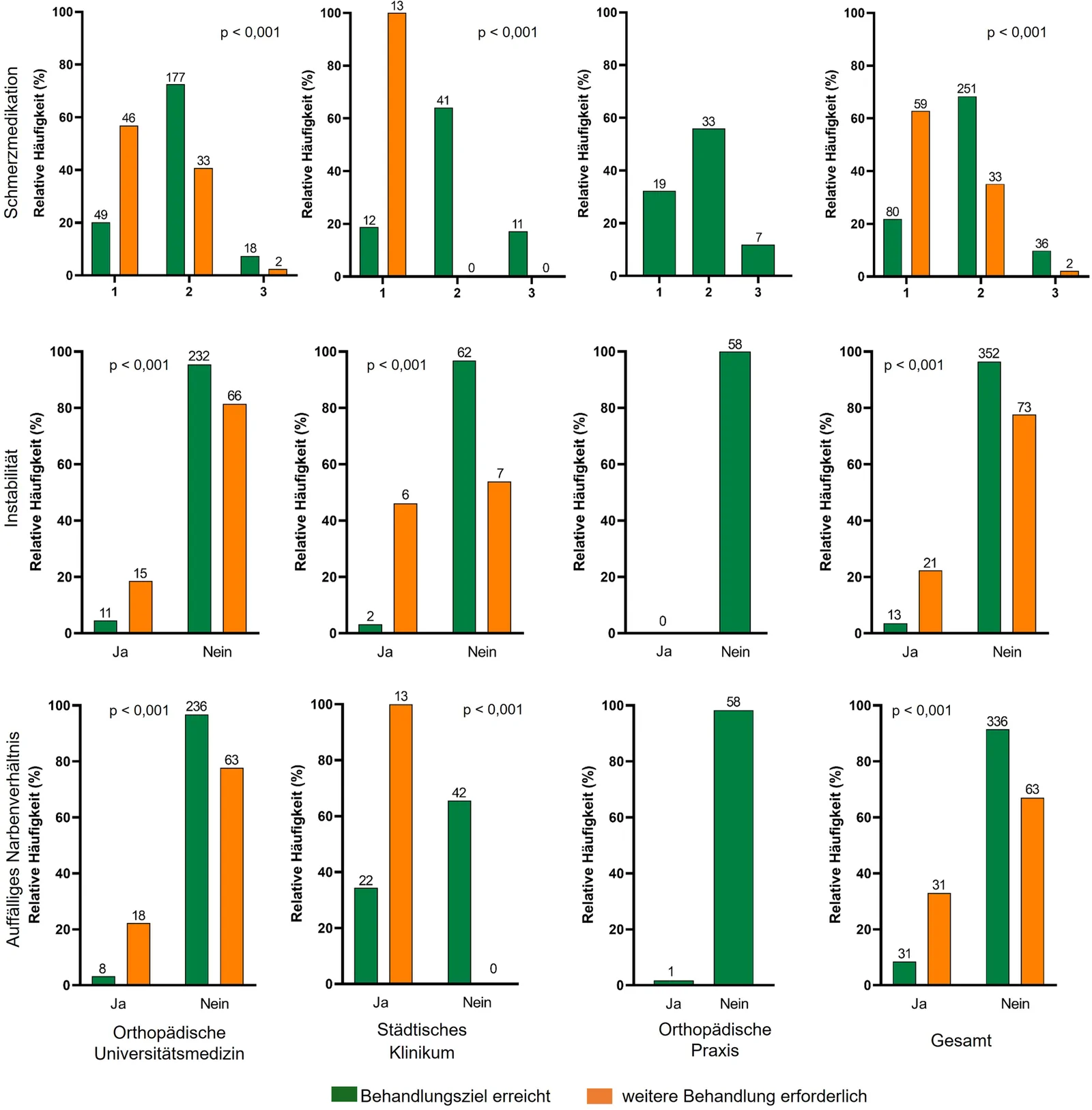
Relative Häufigkeiten ausgewählter Nachuntersuchungsparameter je Zentrumsstruktur sowie zentrumsübergreifend; Schmerzmedikation (1 = ja, wegen des betroffenen Gelenkes, 2 = nein, 3 = unabhängig vom operierten Gelenk)

Postoperative Schmerzen unterschieden sich deutlich zwischen den beiden Behandlungszielgruppen. Patienten, die einer weiteren Versorgung bedürfen, wiesen signifikant höhere Schmerzwerte auf, als jene mit erreichtem Behandlungsziel (U-Test, *p* < 0,001, *p* = 0,006). Dennoch zeigten sich auch in der Gruppe „Behandlungsziel erreicht“ Patienten, die postoperativ Schmerzen vorwiesen (Abb. 2). Zwischen den Studienzentren bestanden keine signifikanten Unterschiede, sodass erhöhte Schmerzen 3 Monate postoperativ unabhängig vom Zentrum mit einem ungünstigen Outcome assoziiert waren (Tab. 3 und 4).

**Tab. 3:** Die ERS-Schmerzintensität und der OKS-Schmerz-Score zur postoperativen Evaluierung eventuell bestehender Schmerzen zwischen den beiden Gruppen „Behandlungsziel erreicht“ und „weitere Behandlung erforderlich“ unterschieden nach den drei Versorgungsstrukturen

Score	Zentrum	Behandlungsziel erreicht			Weitere Behandlung erforderlich			*p*-Wert^B^
		*n* (gültig)	Median (R)	MW (SD)	*n* (gültig)	Median (R)	MW (SD)	
ERS-Schmerzintensität^1^	Universitätsmedizin	244 (225)	3 (0–8)	3,4 (1,9)	81 (79)	5 (0–10)	5 (2,2)	*<* *0,001*^*b*^
	Städtisches Klinikum	64 (46)	4 (0–8)	4 (1,8)	13 (11)	6 (3–8)	5,7 (1,6)	*0,006*^*b*^
	Orthopädische Praxis	59 (41)	4 (0–7)	3,9 (2)	–	–	–	–
	*p‑Wert*^*S*^	0,069^d^			0,255^b^			–
	Gesamt	367 (312)	3 (0–8)	3,6 (1,9)	94 (90)	5 (0–10)	5 (2,2)	*<* *0,001*^*b*^
OKS-Schmerz-Score	Universitätsmedizin	244 (241)	67,8 (14,3–100)	66,3 (17)	81 (81)	50 (3,6–83,3)	51 (18,1)	*<* *0,001*^*b*^
	Städtisches Klinikum	64 (54)	67,8 (32,1–100)	67,7 (16,3)	13 (11)	39,3 (10,7–67,8)	40,9 (15,9)	*<* *0,001*^*b*^
	Orthopädische Praxis	59 (58)	67,8 (21,4–100)	68,9 (16,1)	–	–	–	–
	*p‑Wert*^*S*^	0,652^d^			0,097^b^			–
	Gesamt	367 (353)	67,8 (14,3–100)	66,9 (16,7)	94 (92)	50 (3,6–83,3)	49,8 (18,1)	*<* *0,001*^*b*^

^b^Mann-Whitney-U-Test, ^d^Kruskal-Wallis-Test, *p*-Wert^S^ = Vergleich Studienzentren, *p*-Wert^B^ = Vergleich Behandlungsziel

^1^VAS zur Schmerzintensität, *OKS* Oxford-Knee-Score, *EQ-5D* EuroQol-5D, *n* Anzahl, *R* Range, *MW* Mittelwert, *SD* Standardabweichung, *VAS* Visuelle Analog-Skala

**Tab. 4:** Die Items a und b des ERS zur postoperativen Evaluierung eventuell bestehender Schmerzen zwischen den beiden Gruppen „Behandlungsziel erreicht“ und „weitere Behandlung erforderlich“ unterschieden nach den drei Versorgungsstrukturen

Score	Zentrum	Behandlungsziel erreicht *n* (%)				Weitere Behandlung erforderlich *n* (%)				*p*-Wert^B^
		1	2	3	4	1	2	3	4	
ERS-Item a^1^	Universitätsmedizin	25 (10,3)	164 (67,5)	42 (17,3)	12 (4,9)	4 (4,9)	23 (28,4)	37 (45,7)	17 (21,0)	*<* *0,001*^*a*^
	Städtisches Klinikum	6 (11,3)	33 (62,3)	12 (22,6)	2 (3,8)	0 (0,0)	3 (27,3)	5 (45,5)	3 (27,3)	*0,010*^*a*^
	Orthopädische Praxis	7 (12,1)	39 (67,2)	9 (15,5)	3 (5,2)	–	–	–	–	–
	*p‑Wert*^*S*^	0,983^a^				0,868^a^				–
	Gesamt	38 (10,8)	236 (66,7)	63 (17,8)	17 (4,8)	0 (0,0)	4 (4,3)	26 (28,3)	20 (21,7)	*<* *0,001*^*a*^
ERS-Item b^2^	Universitätsmedizin	71 (29,2)	131 (53,9)	35 (14,4)	6 (2,5)	12 (14,8)	32 (39,5)	29 (35,8)	8 (9,9)	*<* *0,001*^*a*^
	Städtisches Klinikum	17 (31,5)	26 (48,1)	11 (20,4)	0 (0,0)	0 (0,0)	2 (18,2)	6 (54,5)	3 (27,3)	*<* *0,001*^*a*^
	Orthopädische Praxis	20 (34,5)	34 (58,6)	4 (6,9)	0 (0,0)	–	–	–	–	–
	*p‑Wert*^*S*^	0,281^a^				0,104^a^				–
	Gesamt	108 (30,4)	191 (53,8)	50 (14,1)	6 (1,7)	12 (13,0)	34 (37,0)	35 (38,0)	11 (12,0)	*<* *0,001*^*a*^

^a^Chi-Quadrat-Test, *p*-Wert^S^ = Vergleich Studienzentren, *p*-Wert^B^ = Vergleich Behandlungsziel

^1^Hatten Sie während der letzten 4 Wochen beim Gehen Schmerzen in Ihrem betroffenen Gelenk? (1 = nie, 2 = gelegentlich, 3 = häufig, 4 = immer); ^2^Hatten Sie während der letzten 4 Wochen nachts Knieschmerzen, sodass Sie nicht schlafen konnten? (1 = nie, 2 = gelegentlich, 3 = häufig, 4 = immer); *ERS* EndoCert-Risk-Score, *n* = Anzahl

### Assoziation der postoperativen Funktion und Lebensqualität mit der Behandlungszielerreichung in Abhängigkeit von der Versorgungsstruktur

Patienten, die das Behandlungsziel bereits 3 Monate postoperativ erreichten, zeigten signifikant höhere Werte in allen Funktions- und Lebensqualitätsmaßen als Patienten mit weiterem Behandlungsbedarf. Dies betraf sowohl den OKS-Gesamt-Score und -Funktions-Score als auch den EQ-5D-Index und die EQ-5D-VAS (alle Chi-Quadrat-Test, *p* < 0,001). Auch Patienten, die ihren allgemeinen Gesundheitszustand (ERS-Item c) 3 Monate postoperativ als besser einschätzten, erreichten das Behandlungsziel signifikant häufiger, als jene mit unveränderter oder schlechterer Selbsteinschätzung. Zwischen den Studienzentren bestanden hierbei keine signifikanten Unterschiede.

Es sind signifikante zentrumsabhängige Unterschiede in der Gruppe „weitere Behandlung erforderlich“ für jeden der abgefragten Funktions- und Lebensqualitäts-Score zu finden. Es gilt, dass in der Universitätsmedizin höhere, also bessere, Werte in der Gruppe „weitere Behandlung erforderlich“ erreicht werden, als in derselben Gruppe des städtischen Klinikums. Bei Betrachtung des EQ-5D-Index fällt auf, dass in beiden Zentren in der Gruppe „weitere Behandlung erforderlich“ der gleiche Median erreicht wird und sich lediglich der Mittelwert unterscheidet, wonach Patienten der Orthopädischen Universitätsmedizin im Durschnitt eine größere Lebensqualität angaben (Tab. 5).

**Tab. 5:** Der OKS-Gesamt- und -Funktions-Score sowie der EQ-5D-Index und die EQ-5D-VAS zur postoperativen Evaluierung der Funktionalität und Lebensqualität zwischen den beiden Gruppen „Behandlungsziel erreicht“ und „weitere Behandlung erforderlich“ unterschieden nach den drei Versorgungsstrukturen

Score	Zentrum	Behandlungsziel erreicht			Weitere Behandlung erforderlich			*p*-Wert^B^
		*n* (gültig)	Median (R)	MW (SD)	*n* (gültig)	Median (R)	MW (SD)	
OKS-Gesamt-Score	Universitätsmedizin	244 (242)	32 (7–47)	31,4 (7,7)	81 (81)	24 (6–42)	24,4 (8,21)	*<* *0,001*^*b*^
	Städtisches Klinikum	64 (54)	33,5 (18–48)	32,4 (7,5)	13 (11)	19 (5–30)	18,6 (6,7)	*<* *0,001*^*b*^
	Orthopädische Praxis	59 (58)	32,5 (11–48)	32,4 (7,4)	–	–	–	–
	*p‑Wert*^*S*^	0,811^d^			*0,042*^*b*^			–
	Gesamt	367 (354)	32 (7–48)	31,8 (7,6)	94 (92)	23 (5-42)	23,8 (8,23)	*<* *0,001*^*b*^
OKS-Funktions-Score	Universitätsmedizin	244 (241)	65 (15–100)	64,5 (17,5)	81 (81)	45 (15–100)	51,1 (19)	*<* *0,001*^*b*^
	Städtisches Klinikum	64 (54)	65 (35–100)	66 (17,6)	13 (11)	35 (10–55)	35,9 (12,8)	*<* *0,001*^*b*^
	Orthopädische Praxis	59 (58)	65 (20–100)	64,5 (17,9)	–	–	–	–
	*p‑Wert*^*S*^	0,952^d^			*0,016*^*b*^			–
	Gesamt	367 (353)	65 (15–100)	64,9 (17,5)	94 (92)	45 (10–100)	49,3 (19)	*<* *0,001*^*b*^
EQ-5D-Index	Universitätsmedizin	244 (240)	0,887 (0,378–1)	0,848 (0,149)	81 (81)	0,788 (0,11–1)	0,775 (0,18)	*<* *0,001*^*b*^
	Städtisches Klinikum	64 (53)	0,887 (0,378–1)	0,87 (0,121)	13 (11)	0,788 (0,11–0,887)	0,587 (0,288)	*<* *0,001*^*b*^
	Orthopädische Praxis	59 (55)	0,887 (0,175–1)	0,833 (0,174)	–	–	–	–
	*p‑Wert*^*S*^	0,758^d^			*0,038*^*b*^			–
	Gesamt	367 (348)	0,887 (0,175–1)	0,849 (0,15)	94 (92)	0,788 (0,11–1)	0,753 (0,204)	*<* *0,001*^*b*^
EQ-5D-VAS	Universitätsmedizin	244 (242)	75 (6–100)	70,6 (18,1)	81 (80)	65 (8–95)	61,8 (19,1)	*<* *0,001*^*b*^
	Städtisches Klinikum	64 (53)	75 (5–95)	71,1 (17,2)	13 (11)	50 (6–80)	50,1 (18,6)	*<* *0,001*^*b*^
	Orthopädische Praxis	59 (55)	70 (5–100)	65,2 (22,4)	–	–	–	–
	*p‑Wert*^*S*^	0,218^d^			*0,047*^*b*^			–
	Gesamt	367 (350)	75 (5–100)	69,8 (18,7)	94 (91)	60 (6–95)	60,4 (19,3)	*<* *0,001*^*b*^

^b^Mann-Whitney-U-Test, ^d^Kruskal-Wallis-Test, *p*-Wert^S^ = Vergleich Studienzentren, *p*-Wert^B^ = Vergleich Behandlungsziel

*OKS* Oxford-Knee-Score, *EQ-5D* EuroQol-5D, *n* Anzahl, *R* Range, *MW* Mittelwert, *SD* Standardabweichung, *VAS* Visuelle Analog-Skala

### Assoziation der klinischen Nachuntersuchung mit der Behandlungszielerreichung in Abhängigkeit von der Versorgungsstruktur

In der Gruppe „Behandlungsziel erreicht“ fanden sich überwiegend unauffällige klinische Befunde, u. a. eine selbstständige Gehfähigkeit ohne Hilfsmittel, größere maximale Gehstrecken und ein unauffälliges Gangbild. In der Orthopädischen Universitätsmedizin zeigte sich lediglich für das Bewegungsausmaß und in der Beinlängendifferenz kein signifikanter Unterschied zwischen den Gruppen, während alle übrigen Nachuntersuchungsparameter mit der Behandlungszielerreichung assoziiert waren. Im städtischen Klinikum zeigten Bewegungsausmaß, Zohlen-Zeichen und Ruheschmerz keinen signifikanten Zusammenhang mit dem Behandlungsergebnis, wohingegen die übrigen klinischen Parameter signifikant zwischen den Gruppen differenzierten. Der Schmerzmittelbedarf wegen des betroffenen Gelenkes war in der orthopädischen Praxis höher im Vergleich zur Universitätsmedizin und zum städtischen Klinikum (Abb. 2).

Im Folgenden werden schmerz- und funktionsbezogene Parameter der klinischen Nachuntersuchung dargestellt, die u. a. zusätzlich zu den PROM zur objektiven Beurteilung der Behandlungszielerreichung herangezogen wurden.

In der Gruppe „weitere Behandlung erforderlich“ wiesen deutlich mehr Patienten auffällige Narbenverhältnisse (Schwellung/Rötung/Überwärmung) auf. In der Orthopädischen Universitätsmedizin waren es in dieser Gruppe 22 % der Patienten im Vergleich zu 3 % in der Gruppe „Behandlungsziel erreicht“, während in der orthopädischen Klinik sogar 100 % der Patienten in der Gruppe „weitere Behandlung erforderlich“ auffällige Narbenverhältnisse zeigten. Eine Instabilität des operierten Kniegelenks wurde in der Universitätsmedizin bei 18 % der Patienten mit weiterem Behandlungsbedarf dokumentiert, verglichen mit 4,5 % in der Gruppe „Behandlungsziel erreicht“, während bei Gelenkinstabilität im städtischen Krankenhaus 46 % der Patienten mit weiterem Behandlungsbedarf, 3 % der Patienten mit erreichtem Behandlungsziel gegenüberstanden. Zum Zeitpunkt der Nachuntersuchung nahmen 57 % der Patienten mit zusätzlichem Behandlungsbedarf in der Universitätsmedizin weiterhin Analgetika aufgrund von Schmerzen im betroffenen Gelenk ein, gegenüber 20 % in der Gruppe „Behandlungsziel erreicht“, im städtischen Krankenhaus traf dies auf alle Patienten mit weiterem Behandlungsbedarf zu. Über alle Zentren hinweg nahmen 64 % der Patienten mit weiterführendem Behandlungsbedarf weiterhin gelenkbezogen Schmerzmittel ein, verglichen mit 21 % in der Gruppe „Behandlungsziel erreicht“ (Abb. 2).

**Abb. 3:**
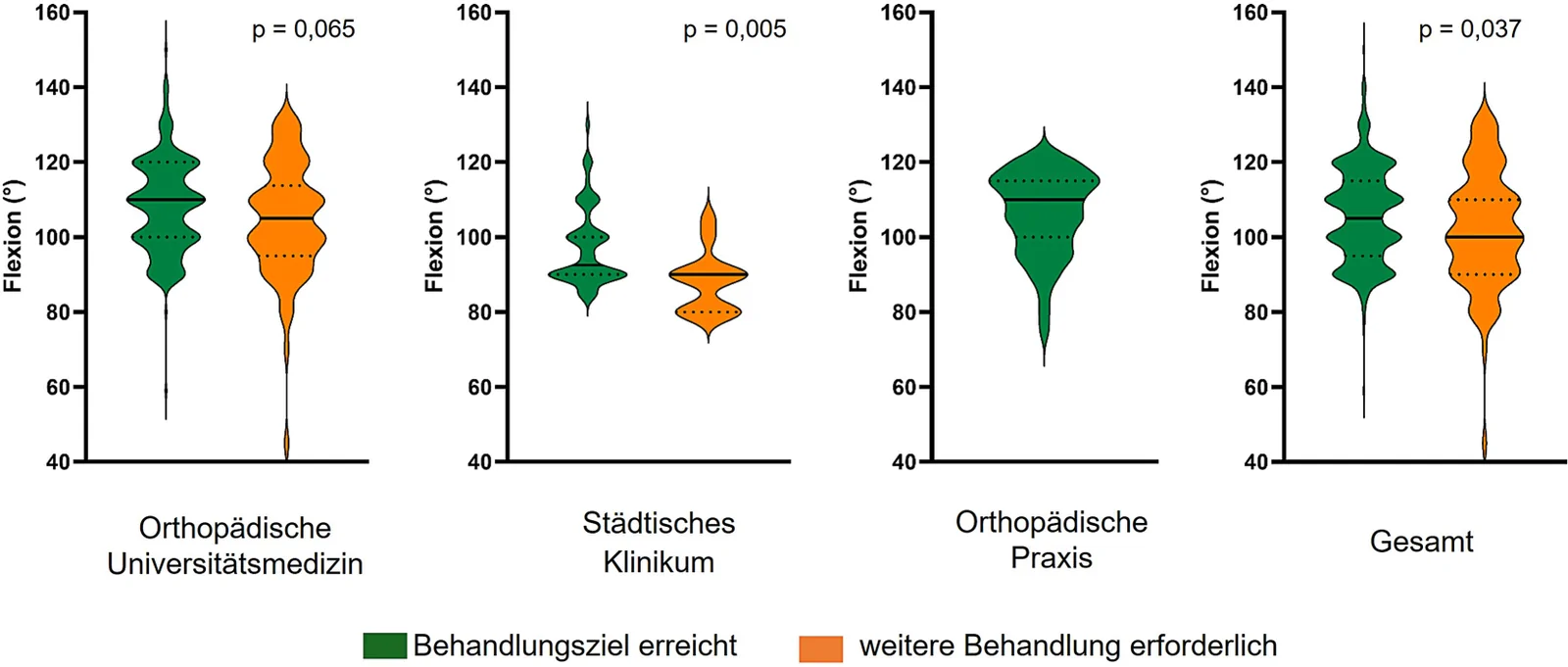
Violinplots der Flexion je Zentrumsstruktur sowie zentrumsübergreifend

Die maximale Kniebeugung war bei Patienten mit erreichtem Behandlungsziel insgesamt höher als bei der Vergleichsgruppe, wobei dieser Unterschied im städtischen Klinikum und in der Gesamtbetrachtung statistisch signifikant war, während sich in der Universitätsmedizin nur ein Trend zeigte (Abb. 3).

## Diskussion

Unsere Ergebnisse zeigen konsistent, dass postoperative PROM eng mit der klinischen Behandlungszielerreichung assoziiert sind. Unabhängig von der Versorgungsstruktur berichten Patienten mit weiterem Behandlungsbedarf 3 Monate postoperativ über signifikant höhere Schmerzen, eine eingeschränktere Funktion und Lebensqualität sowie eine geringere Zufriedenheit als Patienten mit erreichter Zielerfüllung. In Abhängigkeit von der Zentrumsstruktur wurden ebenso Unterschiede nachgewiesen. Während schmerzbezogene PROM keine signifikanten zentrumsspezifische Unterschiede aufwiesen, wurden diese in den funktions- und lebensqualitätsbezogenen PROM bei Patienten mit weiterem Behandlungsbedarf deutlich. Unter Berücksichtigung der Limitationen verdeutlicht dieses die Sinnhaftigkeit der Erhebung von PROM im postoperativen Nachbehandlungsregime.

Damit adressiert die Arbeit eine zentrale Forschungslücke im deutschen Versorgungskontext, in dem PROM bislang nur fakultativ erhoben werden und selten systematisch mit klinischen Nachuntersuchungsbefunden verknüpft sind. Die vorliegenden Ergebnisse unterstreichen den hohen klinischen Informationsgehalt von PROM und die Wichtigkeit der stärkeren Integration in die Ergebnisbewertung nach Knie-TEP, denn unsere Ergebnisse der klinischen Nachuntersuchung spiegeln die subjektiven PROM-Ergebnisse wider.

Abgesehen vom erneuerten Charlson-Index [21], welcher in der Universitätsmedizin erwartungsgemäß höher war als in der orthopädischen Praxis, weist unsere Studienpopulation keine Unterschiede zwischen den drei verschiedenen Zentrumsstrukturen bzw. Studienzentren auf, sodass grundlegend, trotz des o. g. Patientenselektionsbias, die Vergleichbarkeit der Ergebnisse gegeben war. Beim Vergleich der patientenspezifischen Daten zwischen den Gruppen mit unterschiedlichem Behandlungsergebnis sind hingegen signifikante Unterschiede erkennbar, da Patienten, die einer weiteren Versorgung bedürfen, jünger waren und in der Universitätsmedizin zudem einen erhöhten BMI hatten. Das ist vergleichbar mit Studien und Registerergebnissen, die ebenfalls zeigten, dass jüngere Patienten nach einer Knie-TEP höhere Komplikations- und Revisionsraten aufwiesen als ältere [2, 5, 12, 26] und ein erhöhter BMI mit einem erhöhten Komplikationsrisiko einhergeht [27, 29].

Ein zentrales Ergebnis dieser Arbeit ist, dass postoperative PROM 3 Monate nach Knie-TEP zuverlässig anzeigen, ob das klinische Behandlungsziel erreicht wurde. Damit zeigen PROM ein bislang im deutschen Versorgungssystem unzureichend genutztes Potenzial als Instrument der Qualitätskontrolle jenseits komplikations- und revisionsbasierter Endpunkte. Eine systematische postoperative PROM-Erhebung im Rahmen zertifizierter EndoCert-Strukturen und deren Abbildung im EPRD könnte es ermöglichen, Patienten mit ungünstigem Verlauf frühzeitig zu identifizieren und gezielt in bestehende Nachsorgestrukturen einzubinden, ohne zusätzliche Überversorgung zu erzeugen. Das EPRD könnte demnach zusätzlich perspektivisch als Frühwarninstrument zur Identifikation von Patienten mit potenziell weiterem Behandlungsbedarf dienen.

Die differenzierte Analyse nach Zentrumsstruktur verdeutlicht jedoch, dass die Übertragung von PROM-basierten Schwellenwerten in ein potenzielles Frühwarnsystem oder im Rahmen der EndoCert-Audits nicht eindimensional erfolgen kann. Neben patientenspezifischen Risikofaktoren wie Alter und BMI, die bereits in dieser Studie als relevant identifiziert wurden, scheint auch die Versorgungsstruktur des EPZ bzw. EPZmax einen Einfluss auf die Interpretation funktions- und lebensqualitätsbezogener PROM zu haben. Vor diesem Hintergrund erscheint es notwendig, sowohl patienten- (das individuelle Risikoprofil berücksichtigend) als auch zentrumsstrukturspezifische Schwellenwerte zu prüfen, um Fehlklassifikationen zu vermeiden. Zukünftige multizentrische Studien mit größeren Fallzahlen sollten diese Zusammenhänge belastbar quantifizieren und zugleich durch eine einheitliche Untersucherqualifikation methodisch absichern, um sie in standardisierte Bewertungsalgorithmen überführen zu können.

### Schlussfolgerung

Unter der Voraussetzung der Definition klarer ärztlicher objektiver Beurteilungskriterien konnte der Nachweis hoher Wirksamkeit von PROM über die drei verschiedenen Versorgungsstrukturen hinweg aufgezeigt werden. EndoCert-Zentren sollten die Erfassung postoperativer PROM verpflichtend nutzen, um über ein PROM-basiertes Frühwarnsystem, perspektivisch über das EPRD, Patienten mit weiterem Behandlungsbedarf in der frühen postoperativen Phase gezielt einer strukturierten Nachsorge zuzuführen. Eine solche PROM-gestützte Ergebnissteuerung hat das Potenzial, vermeidbare ambulante Nachsorge zu reduzieren und sollte daher durch geeignete finanzielle Anreize oder im Rahmen der Leistungsgruppenzuordnung als Beurteilungskriterium vorausgesetzt werden. Die vorliegenden Ergebnisse zeigen zugleich, dass bei der Interpretation funktions- und lebensqualitätsbezogener PROM patienten- und zentrumsstrukturelle Faktoren berücksichtigt werden müssen, um eine valide Identifikation von Risikopatienten zu gewährleisten.

### Limitation

- In der angebundenen orthopädischen Praxis wurden keine Patienten identifiziert, für die weitere Behandlungen 3 Monate postoperativ erforderlich waren, was die Aussagekraft zentrumsbezogener Vergleiche in diesem Setting einschränkt. Als mögliche Ursachen hierfür kommen sowohl ein Untersucher-Bias durch die Nachuntersuchung durch den Operateur selbst als auch der niedrigere Komorbiditätenindex der dort behandelten Patienten in Betracht. Zudem muss die Indikationsstellung zur weiteren Therapie im eigenen Praxisregime berücksichtigt werden.
- Unterschiede in der Untersucherqualifikation und -durchführung stellen eine weitere Limitation dar, was die Zuordnung zur Behandlungszielerreichung und damit indirekt auch die PROM-Ergebnisse beeinflusst haben könnte.
- Der Einfluss postoperativer Komplikationen wurde in der vorliegenden Analyse nicht gesondert berücksichtigt.

## Fazit für die Praxis

- Postoperative PROM („patient-reported outcome measures“) sind ein valides Instrument zur frühzeitigen Identifikation von Patienten mit weiterem Behandlungsbedarf nach Knietotalendoprothese.
- PROM-basierte Bewertungen liefern klinisch relevante Informationen, die über komplikations- und revisionsbasierte Endpunkte hinausgehen.
- Eine gezielte Nachsorgesteuerung auf Basis postoperativer PROM kann helfen, Ressourcen effizient einzusetzen und Überversorgung zu vermeiden.
- Zentrumsstrukturelle Unterschiede, insbesondere bei Funktions- und Lebensqualitäts-Scores, sollten bei der Definition von Schwellenwerten berücksichtigt werden.
- Die Implementierung eines PROM-basierten Meldesystems über bestehende Registerstrukturen (z. B. EPRD) bietet die Chance, die patientenzentrierte Qualitätssicherung in der Endoprothetik nachhaltig zu stärken.

## Data Availability

Die in dieser Studie vorgestellten Daten sind auf Anfrage beim korrespondierenden Autor erhältlich. Die Daten sind nicht öffentlich zugänglich, können jedoch bei Bedarf bei der klinischen Forschungsabteilung der Orthopädischen Klinik und Poliklinik der Universitätsmedizin Rostock angefordert werden.

## Abkürzungen

BMI: Body-Mass-Index
EPRD: Endoprothesenregister Deutschland
EPZ: Endoprothetikzentrum
EPZmax: Endoprothetikzentrum der Maximalversorgung
EQ-5D-3L: Euroqol-5-Dimensionen-3-Level
ERS: EndoCert-Riskscore
G-BA: Gemeinsamer Bundesausschuss
H-Test: Kruskal-Wallis-Test
Knie-TEP: Knietotalendoprothese
MW: Mittelwert
N: Anzahl
OKS: Oxford Knee Score
PROMs: patient-reported outcome measures
R: Range
SD: Standardabweichung
TEP: Totalendoprothese
U-Test: Mann-Whitney-U-Test
VAS: visuelle Analog-Skala
